# Reducing Thirty-Day Readmissions Among Geriatric Patients Through Multidisciplinary Care: A Tertiary Center Experience

**DOI:** 10.3390/healthcare14182991

**Published:** 2026-09-13

**Authors:** Rehab Yusuf Al-Ansari, Safa Hamdan, Kamal Saleh Al Zahrani, Arifa Jamal, Assim Osman, Nayef Al Ahmadi, Walaa Abouelenien, Mona Al Twayan, Nada Rajab Al Zahrani

**Affiliations:** 1 Hematology Unit, Internal Medicine Department, King Fahd Military Medical Complex (KFMMC), Dhahran 31932, Saudi Arabia; 2 General Medicine Unit, Internal Medicine Department, King Fahd Military Medical Complex (KFMMC), Dhahran 31932, Saudi Arabia

**Keywords:** geriatric, 30-day readmission, infection, multidisciplinary

## Abstract

**Background**: Geriatric patients represent a growing proportion of the population, accounting for more than 9% globally. At our study center, admissions to general medical services comprise over 30% of geriatric patients. This study investigated the reasons for 30-day readmissions among geriatric patients and the impact of a multidisciplinary approach on reducing readmission rates. **Methods**: A retrospective, observational study was conducted at a tertiary care center in the Eastern Province of Saudi Arabia. Data was collected on patients aged ≥ 60 years admitted under the general medicine unit between October 2022 and August 2023 (October–December 2022 for baseline, and January 2023–August 2024 for action, intervention and result). Information was extracted from the hospital’s health informatics system (HIS), including demographics, comorbidities, reasons for readmission, post-discharge care, and outcomes. The multidisciplinary approach included nutritional support (nutritional assessment by referral to dietitian), wound care, virtual post-discharge clinics, family meetings, home healthcare, involvement of social services and psychiatry when indicated. The 30-day readmission rate was calculated by dividing the number of geriatric patients readmitted within 30 days of their last admission by the total number of geriatric patients admitted to internal medicine each month, with a benchmark of 11–23% from previous studies. **Results**: Of 650 geriatric patients, N = 100 required 30-day readmission. Among those 100 geriatric patients (mean age 78), 48% were over 80 years old, 41% had dementia, and 67% were bedridden (defined as the patient’s inability to get out of bed, ambulate, or sit upright without assistance). Hypertension (90%), type 2 diabetes mellitus (78%), and renal impairment (50%) were the top comorbidities. Urinary tract infections (24%) and pneumonia (17%) were the leading causes of readmission. Post-discharge interventions included virtual clinics within 1–2 weeks (49%), home healthcare referrals (27%), and regular outpatient follow-up (87%). Despite a mortality rate of 16% during the study period, the 30-day readmission rate was reduced to less than 23% from 40% in the fourth quarter of 2022 (baseline). **Conclusions**: The observed reduction in 30-day readmissions coincided with multidisciplinary care, yet SPC (Statistical Process Control) analysis confirmed process stability, supporting an observational rather than causal interpretation.

## 1. Introduction

### 1.1. Problem Description

At our center in Dhahran, approximately 1300 patients are admitted annually to the general medical wards, excluding subspecialties such as cardiology, nephrology, and neurology. In 2023 alone, 517 admissions involved geriatric patients, accounting for 39% of all general medical admissions. This substantial proportion underscores the growing burden of geriatric care within our facility.

A review of hospital statistics revealed a significant challenge: during the fourth quarter of 2022, the 30-day readmission rate among geriatric patients was up to 40%. Such frequent readmissions not only reflect the complexity of managing geriatric patients with multiple comorbidities and functional limitations but also highlight gaps in continuity of care and post-discharge support.

This alarming trend provided the impetus to launch a structured quality-improvement initiative. The project was designed to monitor readmission causes and implement a multidisciplinary care model aimed at reducing the 30-day readmission rate. The multidisciplinary approach incorporated nutritional support, wound care, virtual post-discharge clinics, family meetings, home healthcare, and involvement of social services or psychiatry when appropriate.

### 1.2. Available Knowledge

The geriatric age group is not well defined; some references label the age of 60 years as a cutoff age, while other references start from 65 years old [1,2,3]. Geriatric patients account for approximately 4.4% of the population in Saudi Arabia, according to national demographic data [4]. The global share of the population aged ≥ 65 increased from 6% in 1990 to 9% in 2019, and is projected to reach 16% by 2050 [5]. The proportion of people in Saudi Arabia aged 60 or older is predicted to be 25 percent of the total population of 40 million by the end of 2050 [6,7]. As the proportion of older people increases, the prevalence of chronic diseases also rises, together with the risk of having two or more chronic conditions (multimorbidity) [8]. Chronic diseases cause medical, social, and psychological problems that limit the activity of geriatric people in the community [9]. Ultimately, admissions to hospital and readmission rates may increase as a result. These are either due to inability to control underlying disease and/or comorbidities or as a consequence of multiple underlying factors such as decreased oral intake, frailty, urine incontinence, falls, delirium, and pressure ulcers [10,11]. Hospital readmissions are associated with unfavorable patient outcomes and high financial burden, especially in geriatric age groups. Furthermore, the Medicare Payment Advisory Commission (Med PAC) estimated the cost of 30-day readmissions at around $15 billion annually [12]. Other studies also showed a positive association of multimorbidity and in-hospital mortality and healthcare utilization/cost outcomes [13,14,15,16]. In this study, we investigated the reasons for the high 30-day readmission rate among the geriatric age group at a tertiary care center in Dhahran, Saudi Arabia, as well as the impact of managing this population through a strict multidisciplinary and post-admission care approach. Unfortunately, there is no specific international benchmark for readmission rates in geriatric patients; however, reported rates from published evidence vary between 11 and 23% [17]. This study focuses on the geriatric age group and seeks to determine how the quality of care provided to this population can be improved, ultimately reducing the 30-day readmission rate.

### 1.3. Rationale

During 2022, we noticed an increased number of readmissions to the hospital per year with 30-day readmission rate up to 40%. This was a motivation to initiate our improvement project to monitor and to reduce the thirty-day readmission rate in the geriatric population admitted to general medical services by tracking the cause and implementing multidisciplinary team/care approaches, aiming for the target of less than or equal to 23% readmission rate for this age group per month during the first half of the year 2023 and to maintain sustainability of the achieved target by August 2024. As the intervention followed an atypically high baseline quarter, the subsequent decline may partly reflect regression to the mean rather than a true intervention effect.

### 1.4. Specific Aims

In this study, we assessed, analyzed and processed corrective interventions aiming to:

1. Reduce the thirty-day readmission rate in the geriatric population to 23% or less from 2023 up to August 2024.
2. Increase post-care follow-up by using virtual clinics within the first 2 weeks post-discharge to 30% by August 2024.

#### Objectives

- The primary objective is to evaluate the change in the 30-day readmission rate among patients aged ≥ 60 years admitted to the general medicine service following implementation of a multidisciplinary inpatient and post-discharge care program.
- The secondary objectives include describing the characteristics and causes of readmission and evaluating the implementation of specific post-discharge interventions.

## 2. Methods

### 2.1. Context

We conducted a retrospective, analytical, observational study at a tertiary care center in the Eastern Province of Saudi Arabia. The medical records of all geriatric patients (60 years and older) who required admission to the hospital under the general medicine unit between 2023 and August 2024 were involved in this study. These excluded non-geriatric cases as well as geriatric cases admitted under other medical services such as nephrology, cardiology or neurology, as well as the critical care or surgical department. October–December 2022 was used as the baseline date for the study, and January 2023–August 2024 for action, intervention and result.

All data was taken from the health informatics system (HIS), including demographic data, reasons for readmission, post-admission care, outcome, and corrective action plan to minimize readmissions (multidisciplinary approach). We also collected data on age range within the geriatric age group, underlying comorbidities, functional status, length of stay in hospital, follow-up and post-admission care, and patient outcome. As we are aware, 30-day readmissions are subject to competing risks, particularly mortality; therefore, we examined readmission patterns with explicit acknowledgment that death removes patients from the risk pool and may influence observed rates. Furthermore, the multidisciplinary approach included nutritional support, wound care, virtual post-discharge clinic, family meeting, home healthcare and involvement of social services and/or psychiatry whenever needed.

Around 650 medical records of geriatric cases between January 2023 and August 2024 were examined; of these, N = 100 cases required readmission within 30 days during the examined period. A retrospective analysis and close chart auditing were applied to monitor compliance to maintain the 30-day readmission rate in the geriatric age group at the targeted benchmark. Thirty-day readmission was defined as any unplanned hospital admission of a geriatric patient occurring within 30 days of a previous discharge. This included unscheduled readmissions through the emergency department or outpatient clinics. Planned readmissions, observation stays, and readmissions to other hospitals where data could not be captured were excluded from this definition. The flow chart of the process and stages of sample inclusion/exclusion is shown in the Supplementary Material S1.

The primary target was to reduce the 30-day readmission rate to ≤23% per month from baseline in the 4th quarter of 2022 (October–December), then during the first half of 2023, and to sustain this improvement through August 2024. By integrating inpatient multidisciplinary care with comprehensive post-discharge planning, the initiative sought to improve patient outcomes, minimize avoidable hospitalizations, and ensure long-term sustainability of quality of care for the geriatric population.

### 2.2. Intervention

**Figure 1:**
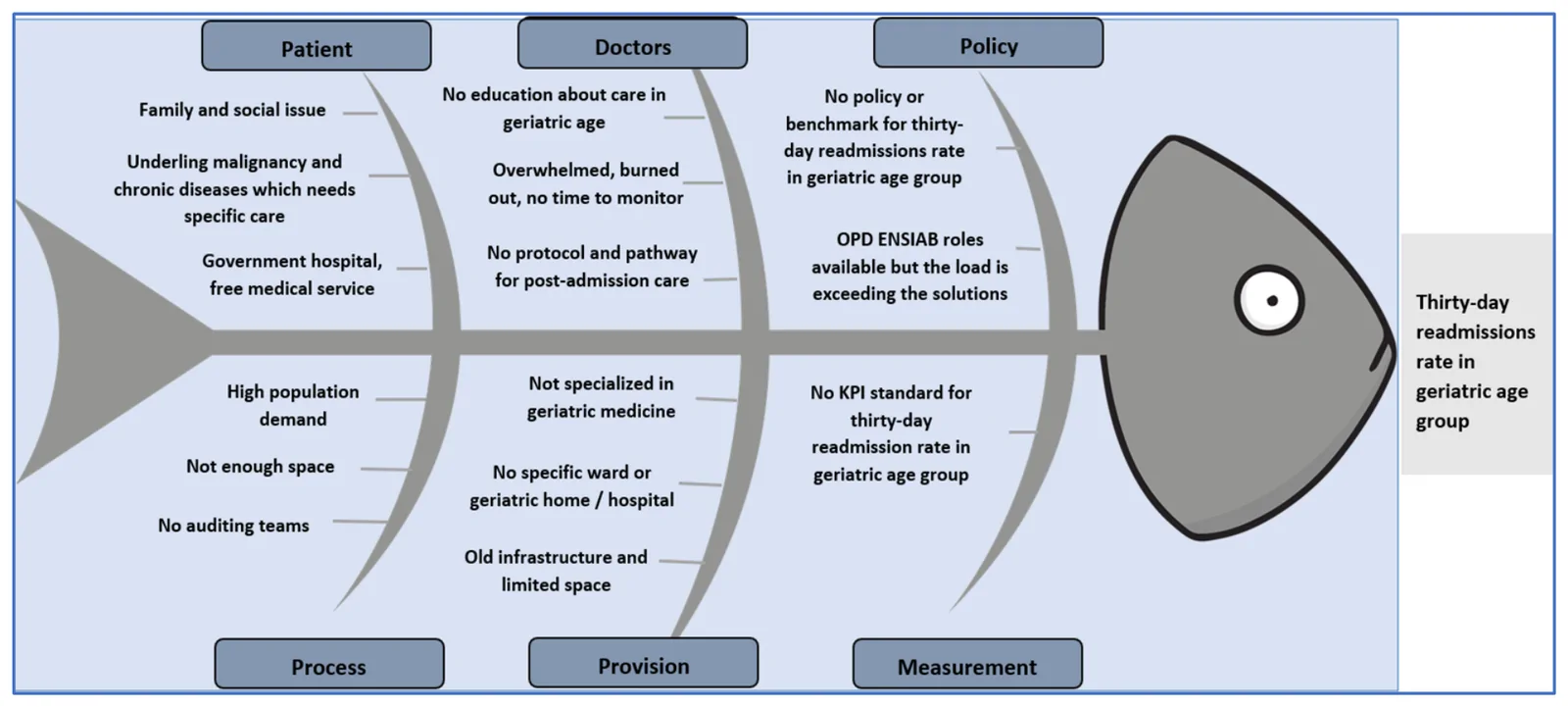
Fishbone view for high rate of 30-day readmissions in the geriatric age group.

To initiate the corrective action plan, we studied the possible reasons behind the 30-day readmission rate in the geriatric population. A fishbone map was established for better understanding of the precipitating factors and to make the correction plan targeted (Figure 1). A corrective action plan team was created for such a purpose.

During 2022, a high rate and frequency of admissions to internal medicine care was noticed. We established an auditing team pre- and post-corrective action plan to minimize the readmission rate among the geriatric age group.

From January 2023 to now, our general medicine doctors have ensured the implementation of multidisciplinary care for geriatric patients as well as comprehensive geriatric assessment (CGA) and intervention and further management based on the result. This entails early assessment and detection of infection (aspiration pneumonia, healthcare-related pneumonia, wound, urinary tract, cellulitis, catheter-related and other sources of infection), wound care, dietitian and feeding requirements, psychiatric and social worker support, family meetings, and education, as well as early involving of subspecialties whenever required and referral to rehabilitation and physiotherapy. By December 2023, a review and a policy of home healthcare were established. However, there were limitations on the cases that could be covered by the home healthcare facility after discharge due to the geographic boundaries for home healthcare as per our hospital’s home care policy. In admission, some of the patients were seen transitionally as they live in other provinces of Saudi Arabia; therefore, education was provided to this category of patient by follow-up at the nearest institute. Further action was carried out in January 2024 with the implementation of virtual clinic within 1–2 weeks post-discharge to ensure safety and good health of geriatric patients. Other actions included widespread education of the general medicine staff and nursing staff in order to optimize patient care. In April 2024, due to a high rate of bed occupancy for end-stage, bedbound cases, the bed management team worked with the medical administration to transfer these patients to rehabilitation centers. By July 2024, outpatient antibiotic therapy (OPAT) was initiated by our colleagues in infectious disease to minimize stay in the hospital for antibiotic management. However, further statistics and auditing are required after this corrective action in the near future to assess its impact. All actions taken are highlighted in Table 1.

### 2.3. Study of the Intervention

Prior to January 2023 (October–December 2022), the rate of readmission and the frequency of admissions per year were obviously high among the geriatric population at the tertiary care center in Dhahran. From 2023 to August 2024, there was no clear data about minimizing the 30-day readmission rate. This was achieved by creating a close chart auditing team, ensuring multidisciplinary care and inpatient early detection of the problem and endorsing early suitable intervention and management with involvement of concerned specialties. Furthermore, comprehensive post-discharge support—including wound care when applicable, routine dietitian referral for all patients, and psychiatry and social service involvement—likely contributed to the improved outcomes observed in our project. Adding to this, ensuring post-discharge care with early virtual clinic implementation, regular OPD and integration of home healthcare services (if within the covering zone, which is up to 25 km from the institute) played a vital role in minimizing the 30-day readmission rate between 2023 and August 2024. Further intervention will take place for a better outcome.

Other measures are described in the measurement Section 2.4.

- The type of measure/indicator was process and outcome.
- The sample size included all geriatric cases admitted to the general medicine unit of any diagnosis at our center in Dhahran including KPI measurement for the percentage of geriatric cases requiring readmission within 30 days, between October 2022 and December 2022 as baseline, and between June 2023 and August 2024 as the intervention and action period.
- Data collection with benchmarking/thresholding reduced the rate of 30-day readmission for patients 60 years old or older by about 23% or less—the previously published benchmark. Data were collected monthly from health information system (HIS) and general medicine inpatient records under the responsibility of the general medicine unit/internal medicine department. Furthermore, the reporting was to the internal medicine department, as well as the Continuous Quality Improvement (CQI) department and patient satisfaction department (PSD).
- During the fourth quarter of 2022, as part of the patient safety and experience project (ENSIAB) evaluation and analysis, the data revealed a high admission and readmission of geriatric patients.
- There had not been any audits conducted to examine the actual statistics of 30-day readmission rate prior to October 2022. This is due to lack of awareness about the need to examine this patient category more closely and to work to minimize readmissions. However, after January 2023, we became aware, and since the geriatric age group is one of the most frequent admission groups in our care, we elected to start our project with this patient group.

### 2.4. Measures

- We measured the rate of 30-day readmission in the geriatric age group per month.
- We measured the percentage of virtual clinic implementation per year.
- We measured the mortality rate per year.

#### 2.4.1. Measuring the Rate of 30-Day Readmission in the Geriatric Age Group per Month

One measure was used that reflects the reduction of 30-day readmission rate: the key performance indicator, measured by applying numerator (N)/denominator (D) values. The calculation is total number of geriatrics with 30-day readmission per month × 100. On the other hand, the denominator (D) was total number of geriatric patients admitted under general medicine per month.

(N) Total number of geriatrics with 30-day readmission per month × 100/(D) total number of geriatric patients admitted under general medicine per month.

**Reason**: It reflects a more accurate percentage of total number of geriatrics with 30-day readmission per month, which is the main purpose of our project.

#### 2.4.2. Measuring the Percentage of Virtual Clinic Implementation per Year

This key performance indicator is measured by applying numerator (N)/denominator (D) values. The numerator (N) was total number of patients with post-admission virtual clinic implementation per year × 100. On the other hand, the denominator (D) was total number of geriatric patients discharged per year.

(N) Total number of geriatric patients with post-admission virtual clinic implementation per year × 100/(D) total number of geriatric patients discharged per year.

**Reason**: As the post-discharge virtual clinic is a new modality of following the patient post-discharge, our aim was to start with a 30% compliance rate and to increase it gradually to reach 100% with the coming year.

#### 2.4.3. Measuring the Mortality Rate per Year

This key performance indicator was measured by applying numerator (N)/denominator (D) values. The numerator (N) was total number of patients with 30-day readmission mortality rate per year × 100. On the other hand, the denominator (D) was total number of geriatric patients discharged per year.

(N) Total number of patients with 30-day readmission mortality rate per year × 100/(D) total number of geriatric patients discharged per year.

**Reason**: Thirty-day readmission is subject to competing risks, particularly mortality and alternative discharge destinations. These events remove patients from the risk pool and may partially explain observed changes in readmission rates.

- Calculation of Control Limits

Control limits help to show expected natural variation in our monthly readmission proportions. These were calculated by measuring the overall proportion (CL: center line).


CL = Total readmissions/Total discharges

Then we calculated the standard error:

SEi = √CL(1 − CL)/ni

where ni is the number of discharges.

Compute the control limits:


UCL = CL + 3 × SEi (upper control limit)

LCL = CL − 3 × SEi (lower control limit)

### 2.5. Data Collection

Data was collected in an Excel sheet, which was obtained from the health informatics system and hospital admission records and stored in a private area with a primary author. Missing information was assessed, and records were not excluded when the absent variables did not affect the primary outcomes.

### 2.6. Analysis

Analysis involved a simple calculation of the percentage (%) and actual number (N). We measured the rate of 30-day readmission in the geriatric age group per month during the study period, the percentage of virtual clinic implementation per year, and the mortality rate per year. Quantity and quality data were analyzed. The graph was drawn from data collected and tabulated in Excel sheets. The graph of the pre- and post-corrective action plan clearly emphasizes the present problem and improvement with sustainability of the result after establishment of the improvement project, which started by taking baseline data between October and December 2022, followed by intervention and auditing of the results on January 2023 and then monthly until August 2024.

### 2.7. Ethical Consideration

There are no unique ethical considerations. Patient consent is not necessary for retrospective anonymized data, and no patient material was used. The study was approved by the Institutional Review Board (IRB) of the Armed Force Hospitals Eastern Region on 7 April 2025, under protocol number AFHER-IRB-2025-006. IRB approval was obtained retrospectively. The data had originally been collected as part of a local quality-improvement initiative aimed at enhancing patient care strategies. Once we elected to publish the findings, formal IRB approval was secured. The study was carried out in accordance with the Declaration of Helsinki.

## 3. Results

**Figure 2:**
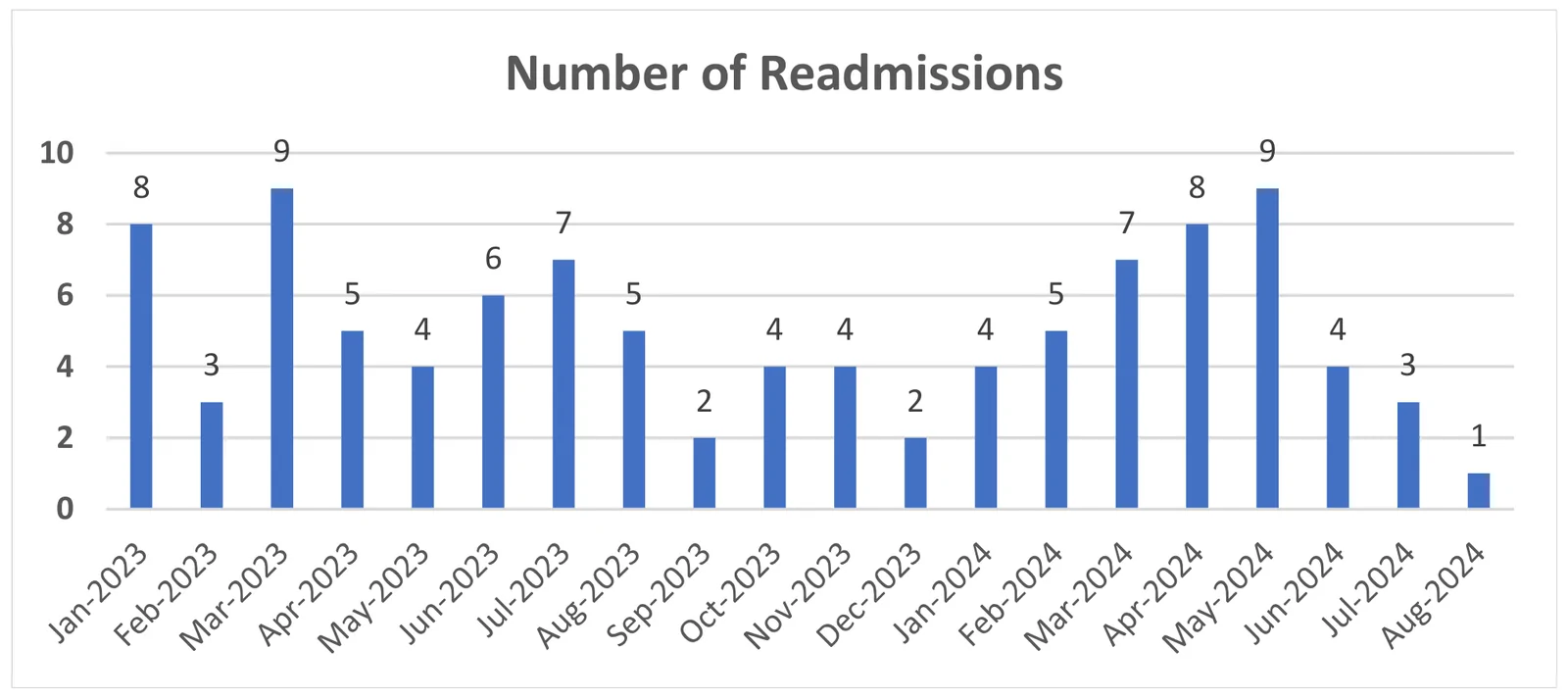
Actual number of 30-day readmissions among the geriatric age group between January 2023 and August 2024.

**Figure 3:**
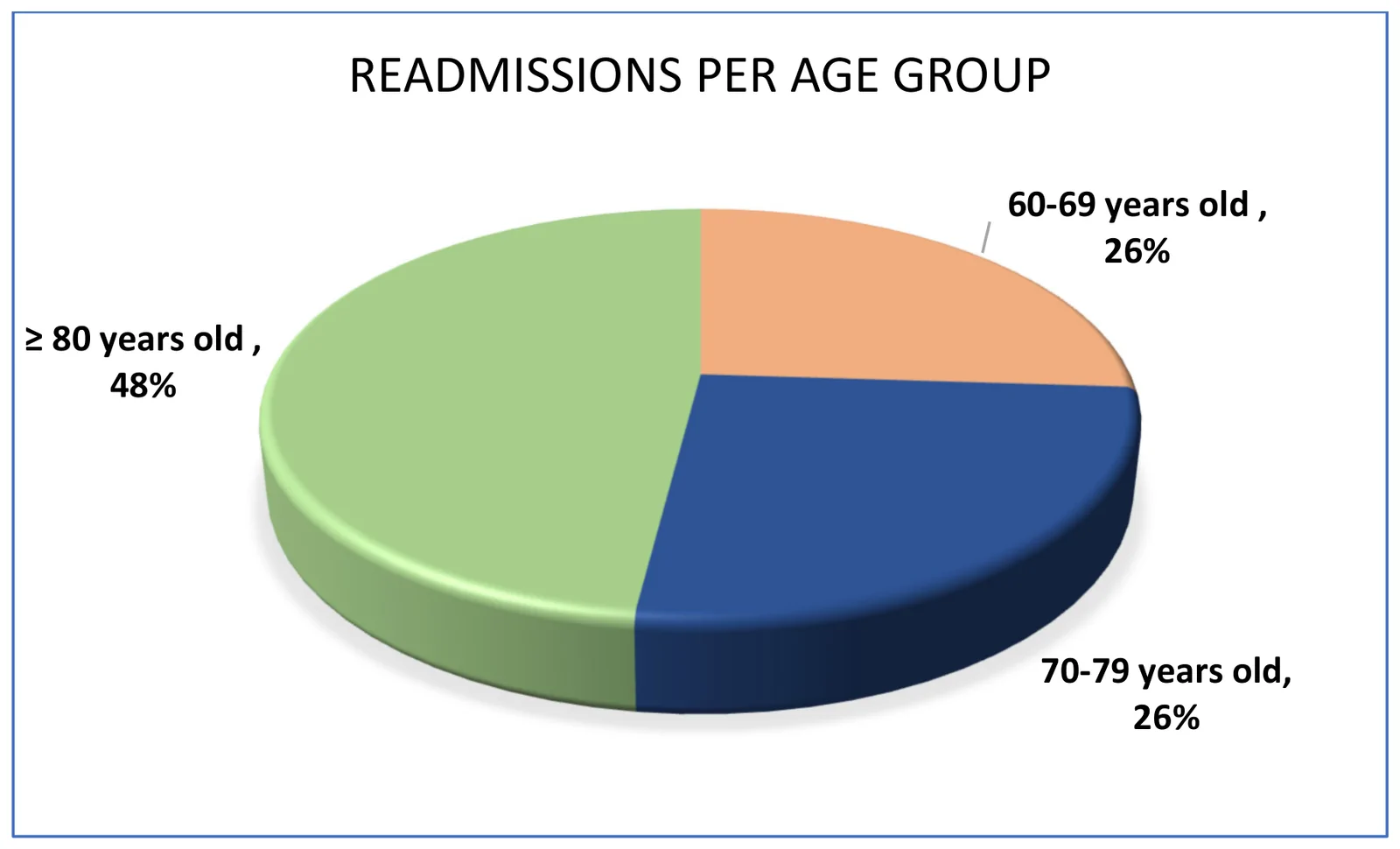
Age distribution among the geriatric patients who required 30-day readmission between January 2023 and August 2024.

We initiated this study after the implementation of the project and collected data retrospectively in the last quarter of 2022 (October–December), then after the initiation of the project between January 2023 and August 2024. A total of 650 files were reviewed; among them, 100 patients were eligible for the study (Supplementary Material S1). The actual number of 30-day readmissions among the geriatric age group between January 2023 and August 2024 is shown in Figure 2. There were up to nine cases with 30-day readmission rate in March 2023 and May 2024. The maximum age was 104 years old and the minimum was 61 years old, with a mean of 78 years old. Around 48% of the patients were over 80 years old, and the remainder of the patients were between 60 and 69 and 70 and 79 (26% and 26%, respectively) (Figure 3). Supplementary Material S2 shows the age categorization with actual number (N) and Standard Deviation (SD).

**Figure 4:**
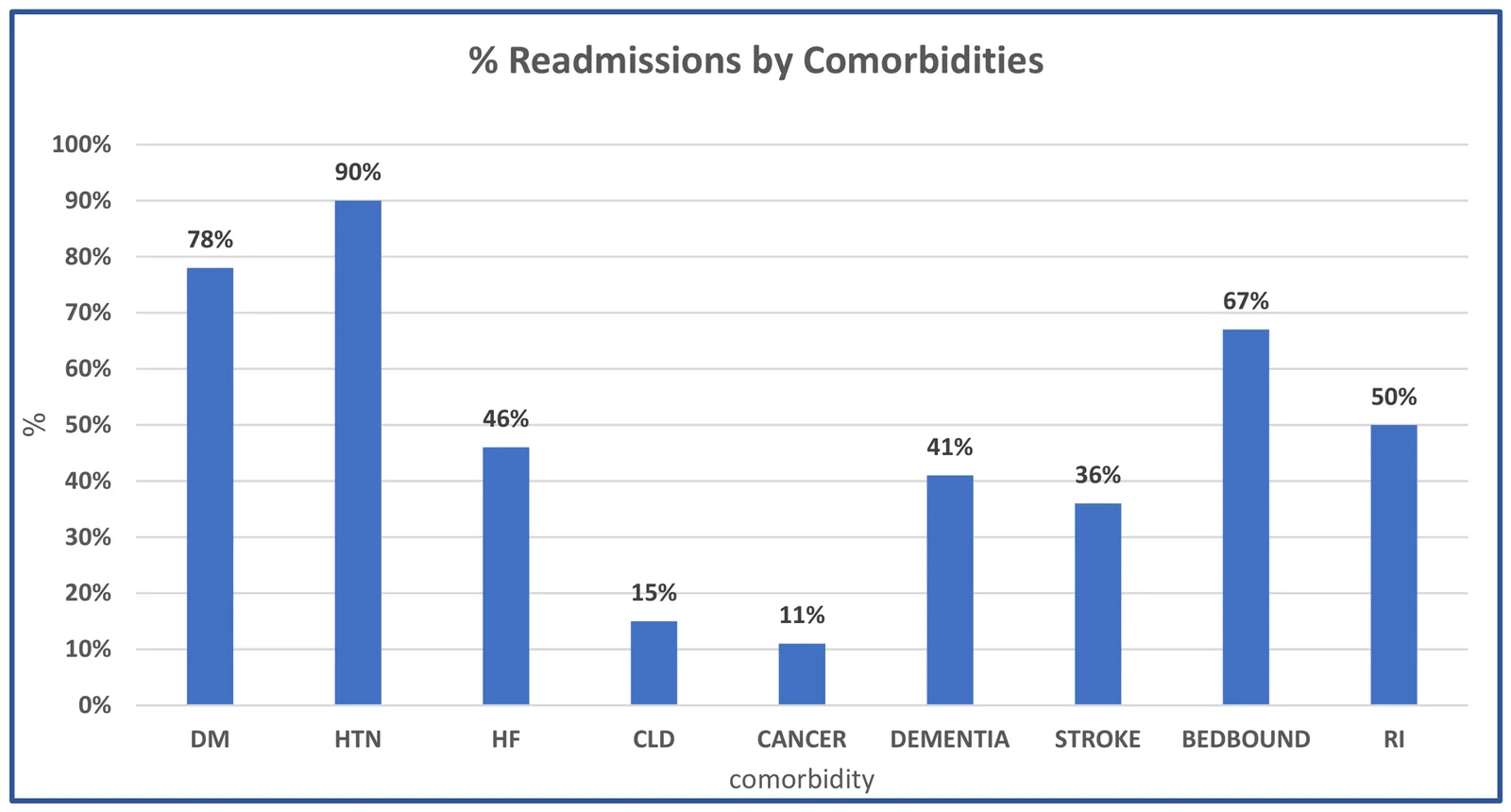
Comorbidities with their prevalence rate among the geriatric patients who required 30-day readmission between January 2023 and August 2024. All comorbidity percentages represent the prevalence among the 100 readmitted patients.

Figure 4 shows the comorbidities and their prevalence rate among the geriatric patients who required 30-day readmission between January 2023 and August 2024. All comorbidity percentages represent the prevalence among the 100 readmitted patients. Hypertension (HTN), type 2 diabetes (DM), and renal impairment (RI) were among the most frequent comorbidities in our study (90%, 78%, and 50% respectively). Renal impairment refers to any reduction in kidney function that affects filtration, electrolyte balance, or metabolic regulation. Among our study sample, 67% were bedbound and 41% had dementia (based on a formal cognitive assessment tool using mini-COG: three-minute test combining three-word recall and a clock-drawing task) (Figure 4).

**Figure 5:**
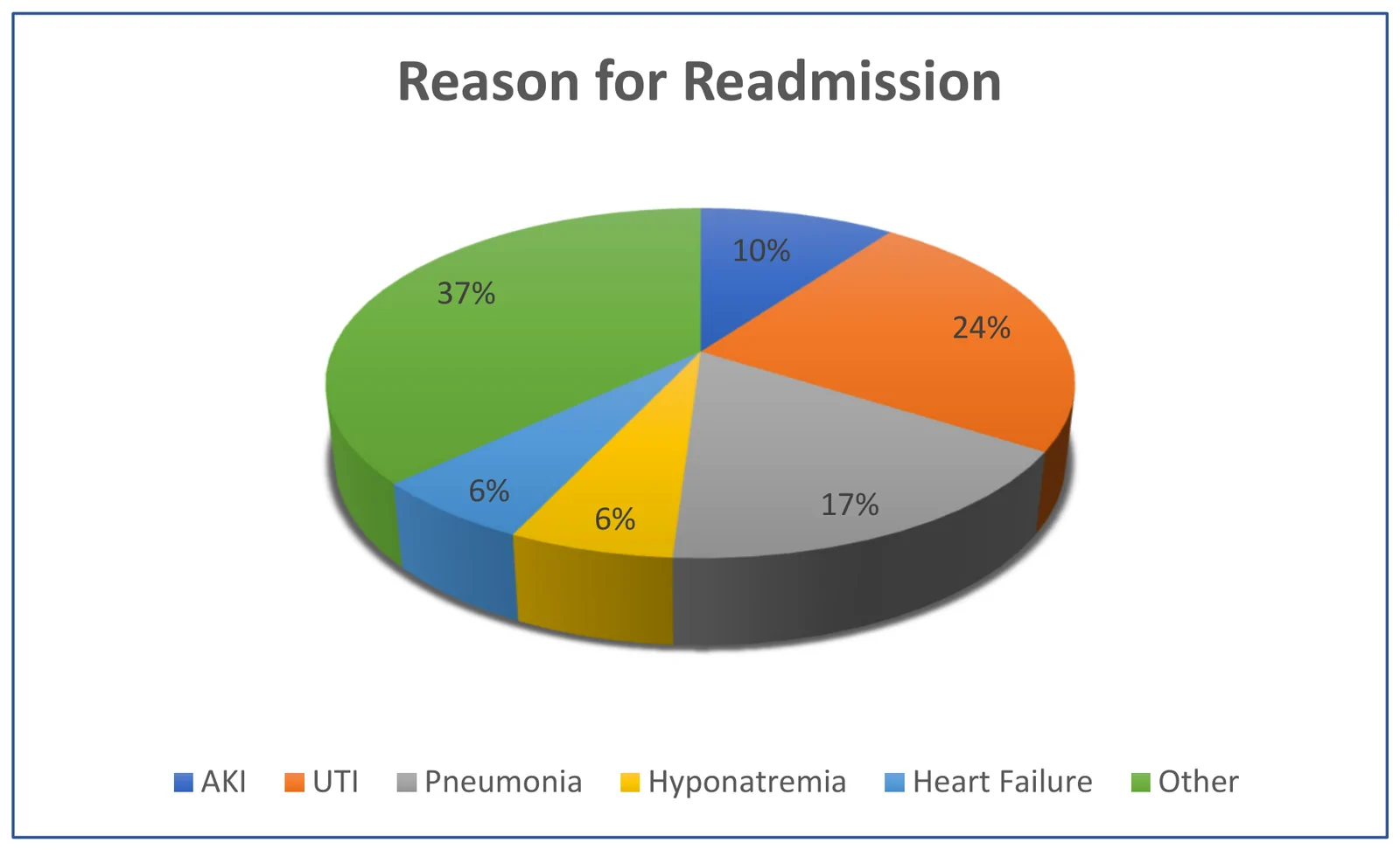
The causes of readmissions among the geriatric patients who required 30-day readmission between January 2023 and August 2024. Other reasons included infections from any other source not mentioned such as gastroenteritis, feeding-tube-related infection or dislodgement, and skin infection, as well as admission for anemia requiring investigation, social reasons, and malignancy and its related complications.

**Figure 6:**
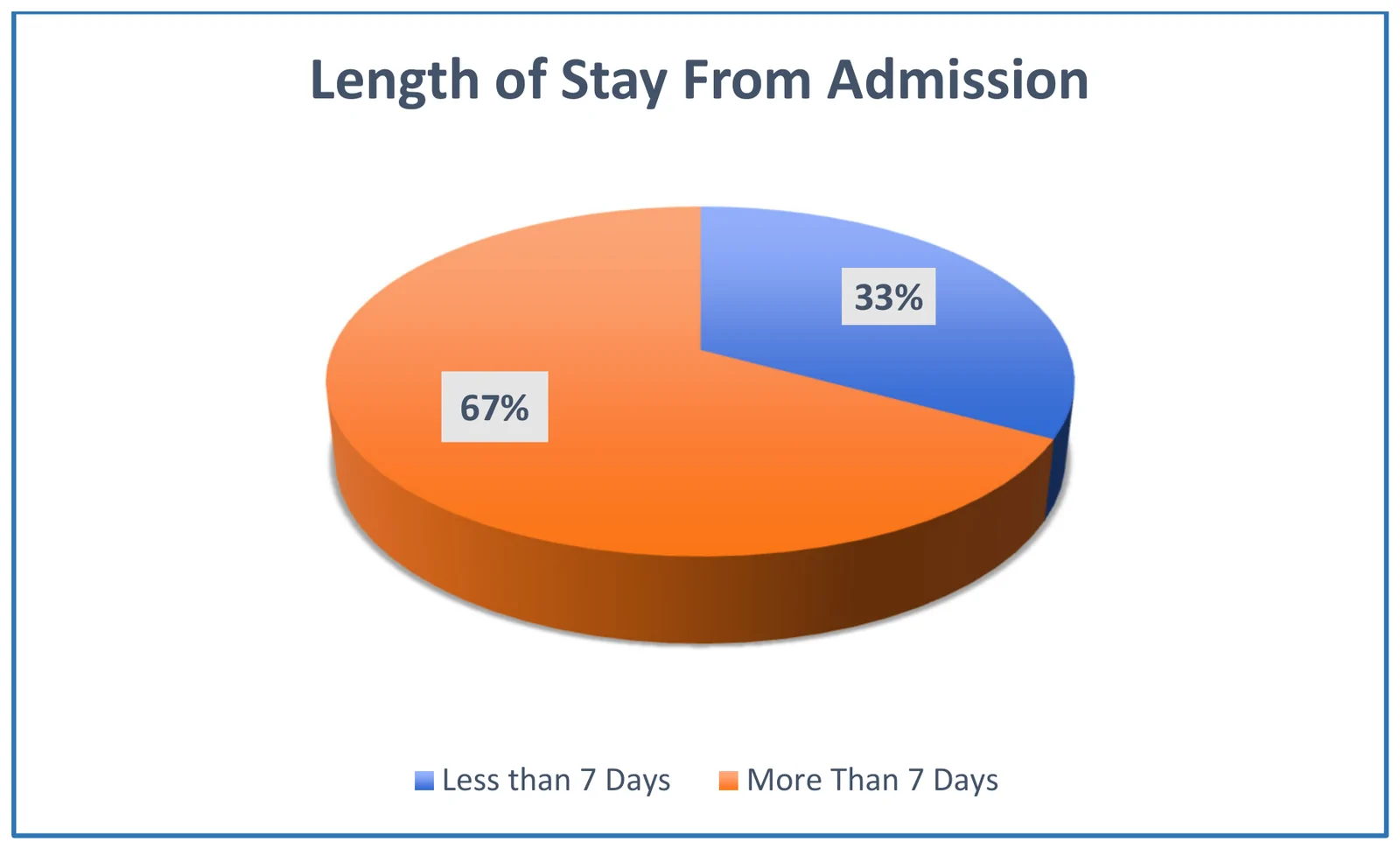
The length of the stay among the geriatric patients who required 30-day readmission between January 2023 and August 2024.

The reasons for readmissions among the geriatric patients who required 30-day readmission between January 2023 and August 2024 were also studied (Figure 5). Taking into consideration that the readmission categories reported in the current cohort are mutually exclusive, with each patient assigned to a single primary cause of readmission, the most frequent causes of readmission were urinary tract infection (UTI), pneumonia of different types (community-acquired, healthcare-acquired, and aspiration), acute kidney injury (AKI), hyponatremia, and heart failure (24%, 17%, 10%, 6% and 6% respectively). The length of the stay among the geriatric patients who required 30-day readmission between January 2023 and August 2024 is shown in Figure 6. Among the 100 patients readmitted, geriatric patients’ length of stay ranged from 1 to 40 days, with a median length of stay during the readmission episode of 9 days (IQR 5–18), with 67% staying more than seven days and 33% staying seven days or less (Figure 6) (Supplementary Material S2).

Table 2 shows the types of referrals, the plan of follow-up after discharge and the outcome among the geriatric patients who required 30-day readmission between January 2023 and August 2024. Dietitian referral and family meetings were done in 100% of the cases during the hospital stay. Wound care was required in 65% of cases, and 39% required psychiatry or social support. On the other hand, the plan of after-discharge care also highlighted giving regular OPD for follow-up in 87% of cases, referral to home healthcare in 27% of cases, and the benefit of virtual clinic 1–2 weeks following discharge in 49% of cases. The mortality outcome was 16% among readmission cases and the remaining cases are either alive based on our hospital records or died in another region/country (Table 2); the readmission/mortality competing-risk framework is available in the Supplementary Material S3.

**Figure 7:**
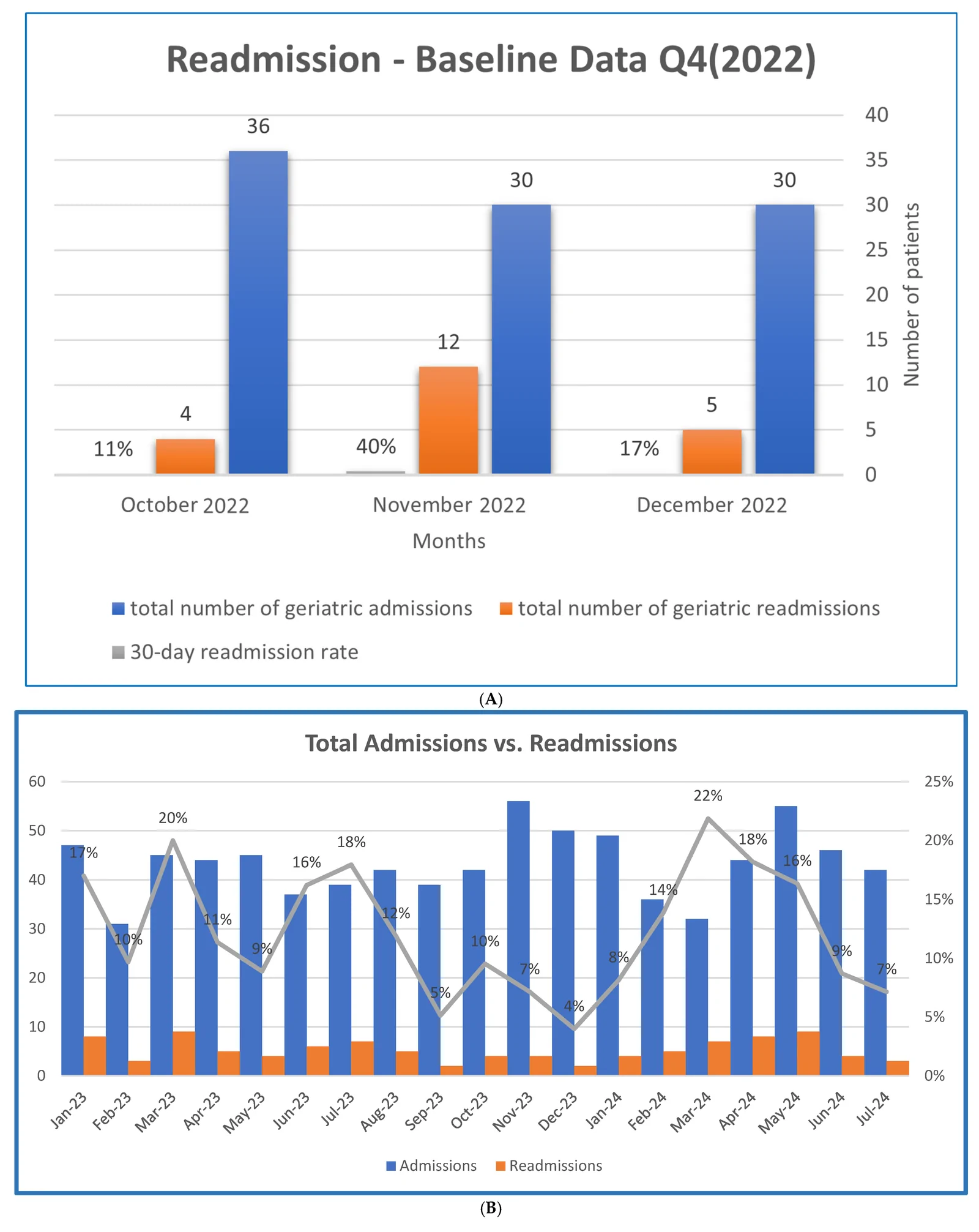

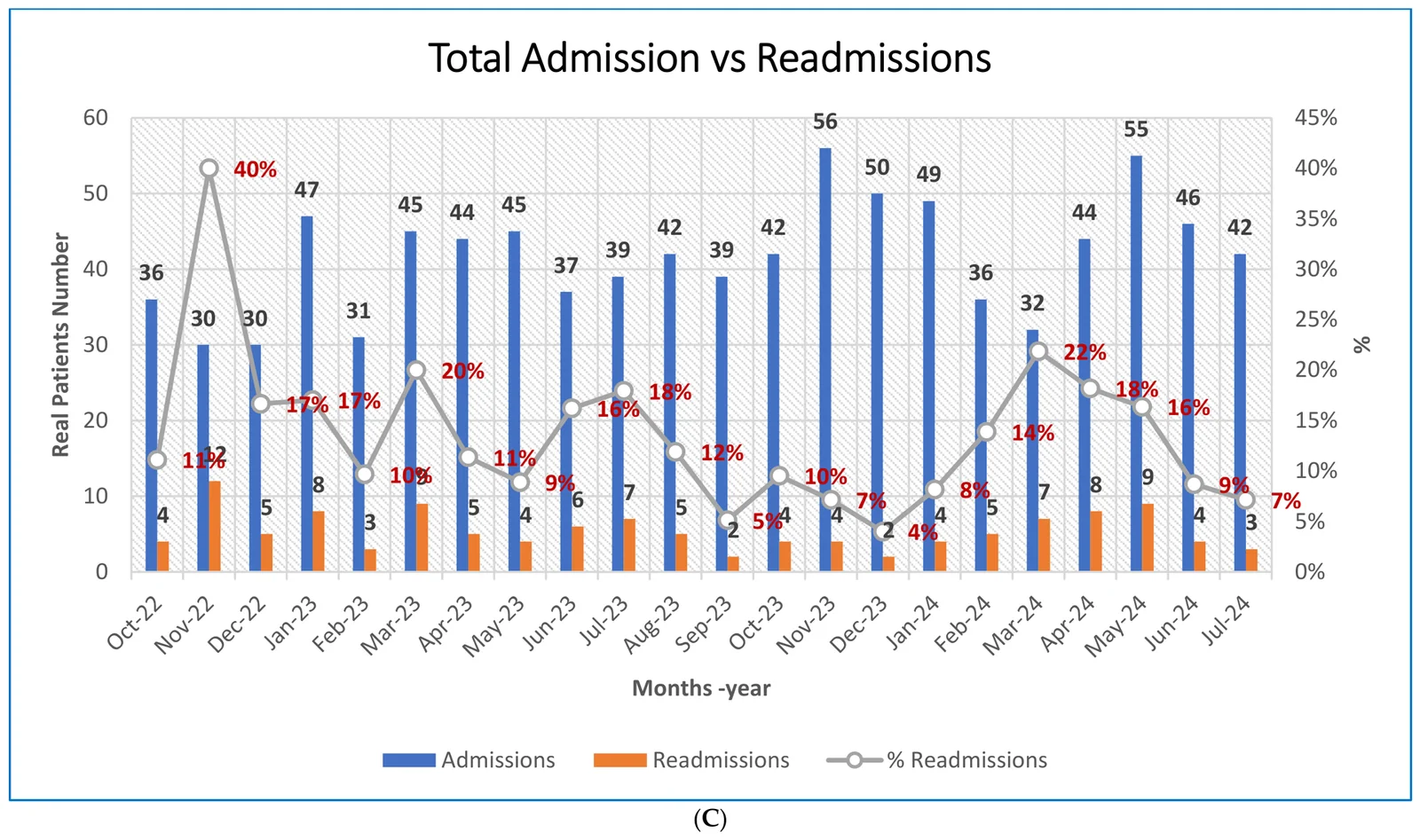
Timeline of the temporal trends of total admission and readmission cases among geriatric the age group (**A**) in the last quarter of 2022, (**B**) then between January 2023 and August 2024. (**C**) Timeline chart through all investigation periods, pre- and post-action plan.

Figure 7 shows the timeline of the temporal trends of 30-day readmission cases and total admission among the geriatric age group in the fourth quarter of 2022 (Figure 7A), then between January 2023 and August 2024 (Figure 7B). It clearly shows the sustainability of maintaining the 30-day readmission rate at less than 23% during such a period, with a more than 17 percentage-point absolute reduction (baseline of 40%). Furthermore, the readmission rate was lowered to 7% in certain months. Figure 7C shows the timeline chart through all investigation periods, pre- and post-action plan, with noticeable changes between the last quarter of 2022 and now.

Monthly 30-day readmission proportions were analyzed using three-sigma control limits. The center line represented the overall readmission proportion (15.4%), and control limits were calculated monthly based on the number of discharges. This approach allowed assessment of process stability and identification of special-cause variation. Across the 22-month period, monthly readmission rates remained within three-sigma control limits, indicating common-cause variation. No special-cause signals were detected following the intervention, and observed reductions were within expected process variability (Supplementary Material S2).

## 4. Discussion

Due to improvements in healthcare services and community outreach programs, life expectancy has increased, resulting in the geriatric population comprising a significant proportion of patients. Consequently, hospital readmissions within 30 days have become an important concern for healthcare systems, particularly among the geriatric population.

Readmissions are strongly associated with increased resource utilization, higher healthcare costs, and poorer patient outcomes. Among hospitalized populations, geriatric patients represent one of the most frequent groups requiring readmission within 30 days of discharge. Those with multiple chronic comorbidities are particularly vulnerable, experiencing significantly higher rates of recurrent admissions compared to patients without comorbid conditions [18,19].

Our study clearly reflects this trend, as the majority of readmitted patients had multiple underlying comorbidities. The most prevalent conditions were hypertension, type 2 diabetes mellitus, and renal impairment, which aligns with findings reported in other studies [12,13,14,15]. These results emphasize the critical need for targeted interventions addressing multimorbidity in geriatric patients to reduce avoidable readmissions and improve overall outcomes.

In current study, the data clearly showed that up to nine cases of geriatric patients required 30-day readmission during the months of March 2023 and May 2024. The transient rise in readmissions during this period may be explained by contextual factors. The months in question correspond to peak summer temperatures in the region, a time associated with higher dehydration risk. This interval also overlapped with Ramadan, during which fasting may exacerbate dehydration and metabolic stress. Other concerns included social issues, which were not clearly documented in the medical records, particularly as the study period coincided with the vacation season for most institutions in our country. This may reflect the need for home nursing services in some cases, where families are unable to leave geriatric patients at home without adequate support.

Our cohort showed that urinary tract infections (UTIs) and various forms of pneumonia accounted for 24% and 17% of rehospitalizations, respectively. This differs from the findings in the Leu et al. study, where the common causes of readmission were systemic diseases (43%), infections (27%), and neurologic diseases such as delirium (18%) [18]. Given the variability across studies in populations, definitions, and diagnostic attribution, these figures are presented descriptively rather than as directly comparable to other reports. On the other hand, neurological and functional status is another factor commonly observed among readmitted geriatric patients. However, the study did not include a multivariable model to evaluate independent predictors of readmission. Accordingly, these variables are presented as descriptive characteristics rather than predictors. Furthermore, inadequate coordination among healthcare and social care professionals, as well as poor communication during care transitions, may increase the risk of readmission among older adults with dementia [20]. Jocelyn Carter et al., in their study, proved that nonmedical support systems and general social policy may be required to reduce frequent readmissions [21]. In our project, a reduction was observed following implementation of the quality-improvement program with a reduction in the 30-day readmission rate among geriatric cases from 2023 to August 2024 by applying multi-service supportive care, as well as ensuring post-discharge care.

However, the 30-day readmission rate decreased toward the lower end of the previously reported 11–23% range; however, without a control group or formal statistical testing, this reduction can be interpreted as a descriptive observation rather than evidence of a causal effect. Because the intervention elements were introduced sequentially (home care in December 2023, virtual clinic in January 2024, rehabilitation transfer in April 2024, and OPAT in July 2024), and no concurrent control group existed, the observed improvement reflects the combined effect of these changes rather than any single component. As such, causal attribution should be made with caution.

Our study did not utilize any geriatric assessment tools, such as the comprehensive geriatric assessment (CGA), which has been shown to predict clinical outcomes in older populations [13,14]. CGA-based interventions have also been proven to reduce the mortality [22]. This includes assessments of cognitive function (e.g., Mini-Mental State Examination), gait and mobility, walking ability, and nutritional status. Unfortunately, there was a higher readmission rate among malnourished hospitalized patients as compared to well-nourished patients [23]. Other readmission prediction models, such as the LACE Index and the HOSPITAL Score, have shown limited and statistically insignificant benefits in predicting readmissions among the geriatric population [13,15]. Adopting a validated geriatric assessment instrument could enhance the prediction of 30-day readmission risk in older adults. As a future improvement step, we plan to develop hospital-level guidelines for standardized geriatric assessment and scoring. Integrating such tools into routine practice is a key strategy for minimizing avoidable 30-day readmissions and strengthening geriatric care pathways.

Readmission occurs in about 20% of Medicare discharges after 30 days, with an estimated annual cost of $15–17 billion [12,24]. This illustrates how crucial it is to lower the geriatric population’s readmission rate to minimize hospital-related cost. Additionally, lowering the readmission rate may lower the incidence of hospital-acquired infections and mental illnesses like depression.

Acute Care for the Elderly (ACE) units are designed to identify patients who are at risk of poor outcomes due to hospitalizations and readmissions. They were introduced in the late nineties. It is beneficial to recognize those who are susceptible, release them early, and rehabilitate them to the best possible level of functioning. Another step-down unit is known as the GEM unit. Moving down a notch, the Geriatric Evaluation and Management Unit concentrates on rehabilitation. GEM units are intended for people who are still cognitively capable but may have experienced a decline in function or a fall as a result of a lacunar infarction.

Our proposed future directions include allocating a dedicated number of beds for geriatric patients and establishing specialized units such as Acute Care for the Elderly (ACE) and Geriatric Evaluation and Management (GEM). In addition, digitizing the comprehensive geriatric assessment (CGA) and updating the Frailty Index will enhance efficiency and accuracy in patient evaluation.

Equally important is the development of clear institutional policies for patients who are actively dying. Hospice and palliative care services should be prioritized to reduce unnecessary investigations and procedures, ensuring that care is focused on comfort and dignity. Under a Do-Not-Resuscitate (DNR) framework, interventions would be limited to essential measures such as IV hydration for comfort, with no routine blood draws or invasive procedures.

To achieve this, current policies must be reviewed, amended, and implemented to standardize end-of-life care practices. These measures will help optimize resource utilization, improve patient-centered care, and uphold ethical standards in managing geriatric patients at the end of life.

This study had several limitations. First, it relied on close retrospective auditing and data collection; however, some important information, such as social and family financial support, was unavailable and could not be adequately assessed. Second, because geriatric patients generally have lower immunity and multiple comorbidities, improvements in outcomes and reductions in admission frequency may be limited compared with the current situation. Third, the absence of a geriatric specialty service within our institution limited the scope of our action plan, requiring us to rely on our best available knowledge and existing resources. Also, a comparison between patients who were readmitted and those who were not would be much more informative as a predictor for effectiveness of intervention; however, this was missed. A further additional limitation is the lack of a concurrent control group for better comparison, as well as the short baseline period which could change the study nature and intervention, and the staggered introduction of multiple interventions. Moreover, seasonal variation, evolving case mix, and changes in admission and discharge practices may also have influenced the findings. Readmissions to external facilities may not have been completely ascertained, and the absence of inferential statistical testing further restricts the strength of causal interpretation.

Another limitation is that evolving clinical practices during the study period—such as the staggered introduction of home healthcare, virtual clinics, rehabilitation transfers, OPAT, and bed management strategies—may have influenced outcomes independently of the intervention. This reinforces that the program should be viewed as a complex quality-improvement initiative rather than a single discrete component.

## 5. Conclusions

Geriatric-age-group patients are among the most frequent admissions to the general medicine department in our center. Studies on the quality of care among this group of patients, as in minimizing the 30-day readmission rate, in Saudi Arabia are generally insufficient. Bringing attention to this age population is important for patient safety, reducing hospital stay and readmission rate, and achieving cost-per-bed savings. Our cohort demonstrates that multidisciplinary inpatient care and structured post-discharge planning coincide with a reduction in 30-day readmissions to ≤23% among older adults; however, SPC analysis indicates that this pattern remains observational rather than causal. Given that the findings are observational and originate from a single center, external validation is necessary. Additional local and international studies are required to verify these results and support wider generalizability. Furthermore, an international standard for geriatric 30-day readmission rates is needed to guide future comparisons and research.

## Figures and Tables

**Table 1:** Corrective action plan and progress.

Area of Concern:	Recommendations	Action Taken	Urgency	Progress/Update
As part of the patient safety and experience project (ENSIAB), a high rate of readmission in the geriatric age group was detected	Create a project for improvement	Implement a project to reduce the readmission rate within 30 days in geriatric patients with benchmark of 23%	October 2022	Done
Meeting with general medicine team to identify the issue and to provide education to solve it	Education on multidisciplinary care for geriatric patients	Meeting for education and comprehensive geriatric assessment	Beginning of January 2023	Done
Multidisciplinary meeting documentation and assessment of geriatric patients	Multidisciplinary meeting must be done for all admitted patients	Policy review to be done each multidisciplinary meeting every 7 days during patient admission and documented in HIS form	End of January 2023	Done
Dietitian/nutrition support	Education on dietitian referral of each admitted patient	Review the policy and document in HIS and the feedback of dietitian/nutrition support	March 2023	Done
No wound care education to the admitted patient/care giver	Initiate the education for wound care and specify round on patients every other day	Education on wound care for patient and care giver	June 2023	Done
Social worker involvement	Education for social workers to evaluate each geriatric admitted patient and solve social issues before discharge	Create form for social worker in HIS to document the assessment	August 2023	Done
No active home care	Establish policy of home healthcare	Review and establish home healthcare policy	December 2023	Done
No active virtual clinic	Create one virtual clinic for each consultant	Initiate virtual clinics	January 2024	Done
Physiotherapy/chest physiotherapy/tracheostomy care	Provide education for physiotherapy/chest physiotherapy/tracheostomy care	Establish the service with help of home care visits	March 2024	Done
No transfer to rehabilitation centers	Establish transfer to rehabilitation center by the help of bed management team	Create detailed medical report with specific needs of each patient to facilitate transfer to rehab center	April 2024	Done
No outpatient antibiotic therapy system available (OPAT)	OPAT to start	Initiation of OPAT by infectious unit	July 2024	Done

**Table 2:** Referrals, follow-up and outcomes among the geriatric patients who required 30-day readmission between January 2023 and August 2024.

January 2023 to August 2024								
Referrals				Follow-Up				Outcome
	Dietitian	Family Meeting	Wound Care	Psychiatry or Social Support	Home Healthcare	Virtual Clinic	OPD *	Mortality
Yes (N (%))	100 (100%)	100 (100%)	65 (65%)	39 (39%)	49 (49%)	27 (27%)	87 (87%)	16 (16%)
No (N (%))	0%	0%	35 (35%)	61 (61%)	51 (51%)	73 (73%)	13 (13%)	84 (84%)
Total N (%)	100 (100%)	100 (100%)	100 (100%)	100 (100%)	100 (100%)	100 (100%)	100 (100%)	100 (100%)

* OPD: outpatient clinic.

## Data Availability

The raw data supporting the conclusions of this article will be made available by the authors on request. The data are governed by institutional and data protection rules.

## Supplementary Materials

The following supporting information can be downloaded at https://www.mdpi.com/article/10.3390/healthcare14182991/s1, Material S1: Flow diagram for inclusion/exclusion process. Material S2: Geriatric Age categories, frequency of admission and length of stay analysis. Material S3: Re-admission/Mortality Competing-Risk Frame. Material S4: Calculate Control Limits.

## References

[1] Lee S.B., Oh J.H., Park J.H., Choi S.P., Wee J.H. (2018). Differences in youngest-old, middle-old, and oldest-old patients who visit the emergency department. Clin. Exp. Emerg. Med..

[2] Houben P., Bormann E., Kneifel F., Katou S., Morgül M.H., Vogel T., Bahde R., Radünz S., Pascher A., Schmidt H. (2022). How old is old? An age-stratified analysis of elderly liver donors above 65. J. Clin. Med..

[3] Singh S., Bajorek B. (2014). Defining ‘elderly’ in clinical practice guidelines for pharmacotherapy. Pharm. Pract..

[4] Basheikh M.A., Balubaid H. (2021). Kingdom of Saudi Arabia. Int. J. Ageing Dev. Ctries..

[5] United Nations (2020). World Population Ageing 2019. UN DESA Population Division. https://www.un.org/en/global-issues/ageing.

[6] Bloom D.E., Canning D., Fink G. (2010). Implications of population ageing for economic growth. Oxf. Rev. Econ. Policy.

[7] Elderly Statistics Publication (2025). General Authority for Statistics. https://www.stats.gov.sa/documents/20117/2435281/Elderly+Statistics+Publication+2025+EN+%281%29.pdf/9d7da20d-5c68-71d2-2651-295be023680e.

[8] Raina P., Gilsing A., Mayhew A.J., Sohel N., van den Heuvel E., Griffith L.E. (2020). Individual and population level impact of chronic conditions on functional disability in older adults. PLoS ONE.

[9] Mobbs C. (2019). The Merck Manual of Geriatrics. http://www.merck.com/pubs/mm_geriatrics/sec2/ch2.html.

[10] World Health Organization (2020). Ageing and Health: Fact Sheet. https://www.who.int/news-room/fact-sheets/detail/ageing-and-health.

[11] Al-Amoud M.M., Omar D.I., Almashjary E.N., Alomary S.A. (2023). Morbidity profile among older people at primary health care centers in Saudi Arabia during the period 2012–2020. Saudi Med. J..

[12] Raval A.D., Zhou S., Wei W., Bhattacharjee S., Miao R., Sambamoorthi U. (2015). 30-day readmission among elderly Medicare beneficiaries with type 2 diabetes. Popul. Health Manag..

[13] Sun C.H., Chou Y.Y., Lee Y.S., Weng S.C., Lin C.F., Kuo F.H., Hsu P.S., Lin S.Y. (2023). Prediction of 30-day readmission in hospitalized older adults using comprehensive geriatric assessment and LACE index and HOSPITAL score. Int. J. Environ. Res. Public Health.

[14] Fitriana I., Setiati S., Rizal E.W., Istanti R., Rinaldi I., Kojima T., Akishita M., Azwar M.K. (2021). Malnutrition and depression as predictors for 30-day unplanned readmission in older patients: A prospective cohort study to develop a 7-point scoring system. BMC Geriatr..

[15] Lehnert T., Heider D., Leicht H., Heinrich S., Corrieri S., Luppa M., Riedel-Heller S., König H.H. (2011). Health care utilization and costs of elderly persons with multiple chronic conditions. Med. Care Res. Rev..

[16] Breen K., Finnegan L., Vuckovic K., Fink A., Rosamond W., DeVon H.A. (2020). Multimorbidity in patients with acute coronary syndrome is associated with greater mortality, higher readmission rates, and increased length of stay: A systematic review. J. Cardiovasc. Nurs..

[17] Loutati R., Ben-Yehuda A., Rosenberg S., Rottenberg Y. (2024). Multimodal machine learning for prediction of 30-day readmission risk in elderly population. Am. J. Med..

[18] Lau H.L., Patel S.D., Garg N. (2021). Causes and predictors of 30-day readmission in elderly patients with delirium. Neurol. Clin. Pract..

[19] Krumholz H.M. (2013). Post-hospital syndrome: An acquired, transient condition of generalized risk. N. Engl. J. Med..

[20] Browne B., Ali K., Tabet N. (2024). 1901 Determinants of hospital readmissions in older people with dementia: A scoping review. Age Ageing.

[21] Carter J., Ward C., Thorndike A., Donelan K., Wexler D.J. (2020). Social Factors and Patient Perceptions Associated with Preventable Hospital Readmissions. J. Patient Exp..

[22] Visade F., Babykina G., Puisieux F., Bloch F., Charpentier A., Delecluse C., Loggia G., Lescure P., Attier-Żmudka J., Gaxatte C. (2021). Risk factors for hospital readmission and death after discharge of older adults from acute geriatric units: Taking the rank of admission into account. Clin. Interv. Aging.

[23] Sharma Y., Miller M., Kaambwa B., Shahi R., Hakendorf P., Horwood C., Thompson C. (2017). Malnutrition and its association with readmission and death within 7 days and 8–180 days post-discharge in older patients: A prospective observational study. BMJ Open.

[24] Rubin F.H., Bellon J., Bilderback A., Urda K., Inouye S.K. (2018). Effect of the hospital elder life program on risk of 30-day readmission. J. Am. Geriatr. Soc..

