# Supplementary material for: Reducing Thirty-Day Readmissions Among Geriatric Patients Through Multidisciplinary Care: A Tertiary Center Experience
Source: Healthcare (Basel). 2026 Sep 13;14(18):2991. doi: 10.3390/healthcare14182991 (PMC13606236; doi:10.3390/healthcare14182991)
Supplement: Supplementary file 1 [file healthcare-14-02991-s001.zip › healthcare-4482648-supplementary.pdf]

Supplement file

Material S1: Flow diagram for inclusion / exclusion process:

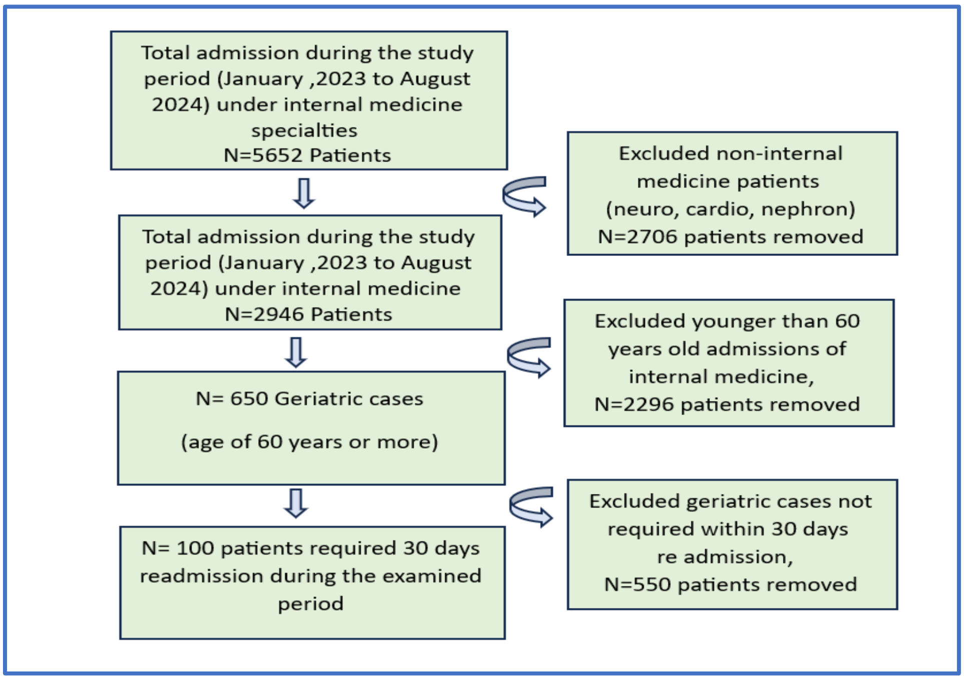

Material S2: Geriatric Age categories, frequency of admission and length of stay analysis

**Geriatric age**	**(%)**	**N (SD)**	
60-69	26%	26 (64.5)	
70-79	26%	26 (74.5)	
Over 80	48%	48 (85)	
**Grand Total**	**100%**	**100**	
**Frequency of admission**			
**Episodes of re admission**	**Frequency (N) / Times**		
2	33		
3	29		
4	22		
5	19		
6	12		
7	5		
**Length of stay for readmission episodes**			
**Days**	**Frequency**	**N**	**(%)**
1 – 4	12	40	33%
5 - 7	28		
8 -10	26	80	67%
11- 13	18		
14 -18	17		
19 - 25	15		
26- 40	4		

Keynotes: percentage (%), number of cases (N) and stander deviation (SD).

Material S3: Re-admission / Mortality Competing-Risk Frame:

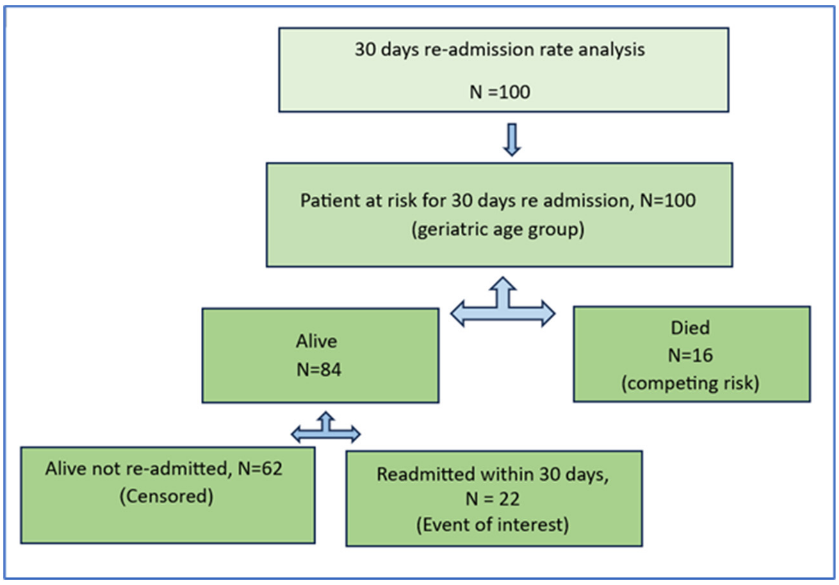

Material S4: Calculate Control Limits.

Month	n	x	p	UCL	LCL
Oct-22	36	4	0.111	0.329	0.000
Nov-22	30	12	0.400	0.361	0.000
Dec-22	30	5	0.167	0.361	0.000
Jan-23	47	8	0.170	0.314	0.000
Feb-23	31	3	0.097	0.356	0.000
Mar-23	45	9	0.200	0.320	0.000
Apr-23	44	5	0.114	0.322	0.000
May-23	45	4	0.089	0.320	0.000
Jun-23	37	6	0.162	0.336	0.000
Jul-23	39	7	0.179	0.331	0.000
Aug-23	42	5	0.119	0.326	0.000
Sep-23	39	2	0.051	0.331	0.000
Oct-23	42	4	0.095	0.326	0.000
Nov-23	56	4	0.071	0.299	0.000
Dec-23	50	2	0.040	0.309	0.000

Jan-24	49	4	0.082	0.311	0.000
Feb-24	36	5	0.139	0.329	0.000
Mar-24	32	7	0.219	0.350	0.000
Apr-24	44	8	0.182	0.322	0.000
May-24	55	9	0.164	0.301	0.000
Jun-24	46	4	0.087	0.315	0.000
Jul-24	42	3	0.071	0.326	0.000

Keynote: **P**: monthly readmission rate (proportion) ; **x**: monthly readmissions; **n**: number of admissions; **UCL**: upper control limit; **LCL**: lower control limit.
